# Orbital magnetism through inverse Faraday effect in metal clusters

**DOI:** 10.1515/nanoph-2024-0352

**Published:** 2024-09-16

**Authors:** Deru Lian, Yanji Yang, Giovanni Manfredi, Paul-Antoine Hervieux, Rajarshi Sinha-Roy

**Affiliations:** Universite Claude Bernard Lyon 1, CNRS, Institut Lumière Matière, UMR5306, F-69100, Villeurbanne, France; Université de Strasbourg, CNRS, Institut de Physique et Chimie des Matériaux de Strasbourg, UMR 7504, F-67000 Strasbourg, France

**Keywords:** magneto-optical response, inverse Faraday effect, time-dependent density- functional theory, plasmon, orbital angular momentum, laser induced magnetism

## Abstract

In view of the recent increased interest in light-induced manipulation of magnetism in nanometric length scales this work presents metal clusters as promising elementary units for generating all-optical ultrafast magnetization. We perform a theoretical study of the opto-magnetic properties of metal clusters through ab-initio real-time (RT) simulations in real-space using time-dependent density functional theory (TDDFT). Through ab-initio calculations of plasmon excitation with circularly polarized laser pulse in atomically precise clusters of simple and noble metals, we discuss the generation of orbital magnetic moments due to the transfer of angular momentum from light field through optical absorption at resonance energies. Notably, in the near-field analysis we observe self-sustained circular motion of the induced electron density corroborating the presence of nanometric current loops which give rise to orbital magnetic moments due to the inverse Faraday effect (IFE) in the clusters. The results provide valuable insights into the quantum many-body effects that influence the IFE-mediated light-induced orbital magnetism in metal clusters depending on its geometry and chemical composition. At the same time, they explicitly demonstrate the possibility for harnessing magnetization in metal clusters, offering potential applications in the field of all-optical manipulation of magnetism.

## 1 Introduction

Accurate control of the magnetic states in materials is emerging as the fastest and the least dissipative method for magnetic writing [1]. Thus, it promises to provide an energy-efficient solution [1], [2] to the bottleneck problem of present-day semiconductor technologies: increased heat dissipation encountered in the miniaturization of storage and computation units. At the same time, developments in controlled and efficient manipulation of ultra-short laser pulses from femtosecond to attosecond timescales has created an impetus to explore ultrafast manipulation of magnetic properties of different materials pushing technological possibilities of faster all-optical switching of magnetization (AOSM) [3], [4], [5], [6], [7], [8], [9], [10]. These recent developments have not only resurrected [2] but also escalated research initiatives [11], [12], [13], [14], [15], [16], [17], [18], [19], [20], [21], [22] on a long discovered physical phenomenon, namely, the inverse Faraday effect [23], [24] (IFE) as it is one of the possible pathways to enable fast AOSM. IFE is a phenomenon where the transfer of angular momentum from a circularly or elliptically polarized light leads to the creation of magnetization in a material.

Due to the strong local enhancement of the electric field at plasmon resonance plasmonic materials are well suited for exploiting the IFE-induced magnetization even though they are often intrinsically non-magnetic. Moreover, size-dependent modulation of plasmonic resonances in metal nanostructures gives the possibility of AOSM over a wide frequency range. In graphene nanodisks transient magnetic fields as strong as 0.7 T have recently been observed [25] by excitation at plasmon resonance frequency in the terahertz range. While exploiting the plasmon resonance to modify the magnetic state in a gold-nanodisc-on-TbCo-thin-film system Parchenko et al. [22] have found that the use of gold nanodisks enhances the effective IFE induced magnetization even when the conditions for plasmon resonance excitation are not perfectly met. Gonzalez-Alcalde et al., has observed [18] enhanced IFE-induced Faraday rotation at plasmon frequency of gold nano-disks in an array of 50 nm thick gold nano-disks. When excited at plasmon energy, 0.95 *μ*_*B*_/atom IFE-induced magnetic moment was observed in 2-mm thick colloidal suspensions of 100-nm-diameter gold nanoparticle (NP) by Cheng et al. [11]. All these experimental observations confirm that despite significant technical differences in them the IFE-induced magnetization is robust at plasmon resonance.

Since its phenomenological conception by Pitaevskii [23] different theoretical approaches have been employed to explain the IFE in plasmas [26], nano-rings [27], and other materials. Quantum theory of IFE has been proposed using perturbation theory to second order in the radiation field [28], and also for solids described by generic itinerant band [29]. Berritta et al. have studied the effect of spin–orbit coupling on IFE in different bulk materials using density functional theory (DFT) [30]. The spin and orbital contribution to IFE has also been studied using hydrodynamic model of the conduction electron gas [31] for nonmagnetic metals [14]. The plasmonic properties of the IFE in metal NPs were also investigated by several groups using multiphysics modeling [32], finite-difference time-domain method [33], hydrodynamic treatment of electrons [34], [35], and very recently using time-dependent density-functional theory (TDDFT) [36]. Hurst et al. [34], used a quantum hydrodynamic (QHD) model [37] to describe the IFE in gold NPs arising from the excitation by circularly polarized light at the localized surface-plasmon resonance (LSPR) energy. While in this model the electrons are treated as a charged fluid, they lack the orbital resolution. By treating electrons quantum mechanically in NPs described by jellium spheres [36], some of the authors of this article have confirmed that significant orbital magnetism can be successfully triggered through the IFE by irradiating non-magnetic NPs (about 0.04 *μ*_*B*_ per electron in the best case for potassium) at LSPR frequency with circularly polarized light. The robustness of this light-induced magnetism and the many-body effects on it are also discussed over a wide range of parameters, including atomic species, laser intensity, and the size of the NP. The per-atom magnetic moment normalized with respect to the laser intensity and the pulse-width matched the same order of magnitude as found by Cheng et al. [11] for Au NPs.

Motivated by this increased interest in understanding the fundamental mechanisms of IFE and inspired by the miniaturization in current technologies, this work explores for the first time the feasibility of generating light-induced magnetism in metal clusters, which are an order of magnitude smaller than NPs and are beyond the scope of modeling by jellium description. We explore the quantum mechanical nature of the IFE-induced orbital magnetism from a purely ab-initio perspective by explicitly considering atomically precise description of metal clusters within the many-body theoretical framework of the time-dependent density-functional theory (TDDFT). We simulate the time-dependent dynamics of interaction of circularly polarized light with clusters of simple and noble metals containing a few tens of atoms. By considering the quantum many-body effects of interacting electrons, the contribution of the chemical species, and the laser pulse on the generation of orbital magnetic moment through the IFE is investigated by analyzing the time-dependent dynamics of electrons excited by circularly polarized light.

## 2 Methods

### 2.1 Ab-initio theoretical framework

We employed the real-time (RT) approach of TDDFT where the evolution of the many-electron system under an external perturbation is obtained from the solution of the one-particle Schrödinger-like equations known as time-dependent Kohn–Sham (TDKS) equations. They contain an effective time-dependent potential known as Kohn–Sham potential which, by theoretical construction, is a functional of the time-dependent electron density *n*(**r**, *t*), and contains both the Hartree and the exchange-correlation (XC) contributions, thus accounting for the many-body effects. Here we approximate the time-non-local dependence of the Kohn–Sham potential by considering that it is a functional of the instantaneous density. Furthermore, the XC part of the Kohn–Sham potential is not known exactly and, in practice, requires an approximate potential. We have employed the adiabatic gradient-corrected Perdew–Burke–Ernzerhof (PBE) approximation [38], which describes collective excitations well in metal clusters without using extensive computation resources. In addition, to correct the self-interaction error so that the asymptotic behavior of the effective potential can be properly described, a scheme based on the average density [39] is used for the simple metals. The many-electron system is perturbed at its ground state which is calculated in static DFT using the same approximations and corrections for the static KS potential as in the time-dependent one. The absorption spectrum is obtained in RT-TDDFT using the time-propagation formalism of Yabana and Bertsch [40], [41].

### 2.2 Systems and simulation details

**Figure 1:**
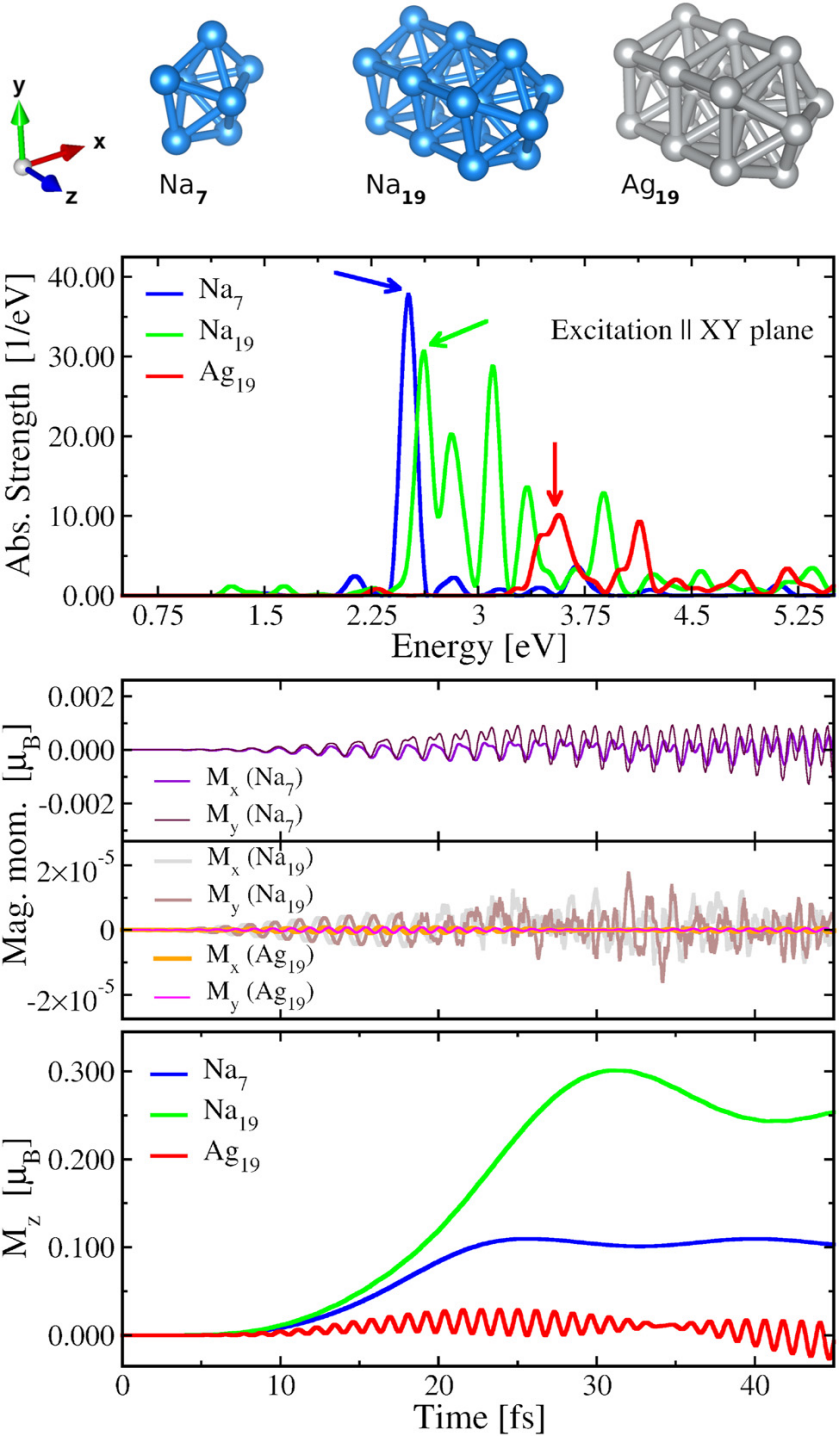
**Top**: the geometrical structures of Na_7_, and rod-like clusters Na_19_ and Ag_19_, and their absorption spectra corresponding to excitation along the lateral direction (i.e. perpendicular to the rod axis: XY plane). The arrows represent strongest resonance in the near-ultraviolet and visible part of the spectra. **Middle**: time dependance of the *x*-and *y*-component of the orbital magnetic moment (i.e. M_*x*_(*t*) and M_*y*_(*t*)) generated due to the excitation by circularly polarized laser pulses with plane of polarization along the XY plane (i.e., same as for the spectra) and frequency corresponding to the LSPR of respective clusters marked by the arrows in the spectra. **Bottom**: M_*z*_(*t*), time dependance of the *z*-component of the generated orbital magnetic moment.

We have considered model clusters of Na and Ag having pentagonal symmetry and both rod-like (Na_19_ and Ag_19_) and disk-like (Na_7_ and Ag_39_) shapes. The seven-atom structure, Na_7_, is derived from the five-atom pentagonal ring of 13-atom decahedral cluster (having *D*_5*h*_ symmetry) and by keeping the two atoms on both side of the ring. This cluster has been well studied before by many experimental groups [42], [43] and the calculated absorption spectrum (cf. top panel Figure 1) is in accordance with earlier results. The 19-atom structures are derived by stacking the same five-atom rings plus a central atom along the five-fold rotational axis (see Figure 1). These kind of geometries have previously been studied for the effect of aspect ratio on absorption spectra of noble-metal nanorods [44], [45], and recently also for electromagnetic screening in metal clusters [46]. The 39-atom structure is derived by surrounding the 19-atom structure with two coaxial ten-atom rings having a diameter twice that of the five-atom rings of the 19-atom structure. The geometry of this structure has partial icosahedral symmetry as shown in Figure 4. Since the structures are treated as model systems, their geometries were not relaxed.

The ionic potential in these clusters is described by atom-centered norm-conserving Troullier–Martins pseudopotentials [47]. The number of valence electrons per atom is considered to be one *s*-electron for Na and one *s*-electron plus ten *d*-electrons for Ag. All calculations are performed in the real-space code octopus [48]. The real-space grid spacing was set to 0.18 Å. The calculation domain was constructed by the union of spheres around each atom of the cluster. The radius of the sphere was set to be 5 Å and 10 Å for atoms in the Ag and Na clusters, respectively. For the time-propagation of the TDKS equations the ETRS propagator (“enforced time-reversal symmetry”) was used. The interval between two consecutive time-steps was set to 0.0024 *ℏ*/eV ( ≈1.58 attoseconds) and 0.0019 *ℏ*/eV ( ≈1.25 attoseconds) for Ag and Na clusters respectively. To obtain the absorption spectra using the *δ*-kick RT-TDDFT method [40], [41] we have used a total propagation time of 37.5 fs. A damping of 0.0037 atomic units of energy (≈ 0.1 eV) was also used for the exponential window function during the Fourier transform. The time-dependent orbital magnetic moment is calculated from the time-dependent current density which are written for every 50th step of time-evolution.

## 3 Results and discussion

### 3.1 Magnetic moment at plasmon resonance

A metal cluster is maximally responsive to an external optical perturbation when excited at the energy of LSPR which is a collective resonant interaction of the conduction electrons with the electromagnetic field. LSPRs are characterized by well-defined peaks in the absorption spectrum. In the top panel of Figure 1 we show the absorption spectra obtained by *δ*-kick RT-TDDFT calculations [40], [41] for Na_7_, Na_19_ and Ag_19_ clusters due to excitation along the *x*-axis. As the clusters are highly symmetric along the XY plane, the spectra along any direction on the XY plane remains the same. We observe the LSPR peak for Na_7_ (the blue curve) at *ω*_*p*_ = 2.53 eV. For the Na_19_ cluster, due to its shape the electrons are more confined in the lateral direction (XY plane) as compared to the axial one (*Z* direction). Due to this quantum (size-)confinement in the XY plane the electronic transitions responsible for the plasmon along this plane split into many energetically close electron-hole excitations giving rise to plasmon fragmentation. This means that the LSPR in the XY plane consists of several energetically close transitions (the green curve). The strongest of these transition is at *ω*_*p*_ = 2.65 eV. Finally, in Ag_19_ (the red curve) we observe broad peaks, the strongest is centered around *ω*_*p*_ = 3.54 eV. This broadening is because of strong coupling between the fragmented LSPR peak and the excitation arising from interband transition involving *d*-electrons of Ag atoms. In order to generate strong orbital magnetic moment we have excited the metal clusters with circularly polarized laser field which can, in general, be described as.

(1) $$E\left(t\right)=E_{x}\left(t\right)\hat{x}+E_{y}\left(t\right)\hat{y}=F\left(t\right)\left[\cos\left(\omega_{L}t\right)\hat{x}+\cos\left(\omega_{L}t-\frac{\pi }{2}\right)\hat{y}\right]$$

where,

(2) $$F\left(t\right)=E_{0}\left\{\begin{array}{cc} & \sin^{2}\left(\frac{\pi }{2\tau_{0}}t\right)\;for\;t\leq \tau_{0};\\ & 1,for\;\tau_{0}<t\leq t_{uni},\;where\;t_{uni}=n_{uni}\tau_{0};\\ & \cos^{2}\left(\frac{\pi }{2\tau_{0}}\;\left(t-t_{uni}\right)\right),for\;t_{uni}<t\leq t_{end},\\ & \;where,\;t_{end}=t_{uni}+\tau_{0};\\ & 0,\;for\;t>t_{end}, \end{array}\right.$$

where *τ*_0_ is chosen to be 20*ℏ*/eV (≈13 fs), and *ω*_*L*_ represents the frequency of the laser field. The maximum amplitude is $E_{0}=10^{-2}\;V/\overset{{}^{\circ}}{A}$ ; *t*_uni_ = *n*_uni_ *τ*_0_, where *n*_uni_ is an integer and determines the constant-in-time segment of the envelope. The duration of the ascending segment of the pulse is *τ*_0_, the same is for the descending segment, and the duration of the uniform segment is (*n*_uni_ − 1)*τ*_0_, which makes the total duration of the laser pulse (*n*_uni_ + 1)*τ*_0_. The temporal profile of the laser pulse overlaid with the piece-wise envelope function is shown in the Figure S2 of the Supplementary Material. As shown in the Equation (1), the plane of polarization of the electric field is along the XY plane (i.e., same as for the spectra). The time-dependent orbital magnetic moment is calculated as,

(3) $$M\left(t\right)=\frac{1}{2}\underset{V}{\int }dr\;r\times j\left(r,t\right)$$

where, *V* is the volume of the simulation domain, **r** is the displacement vector from the center of mass of the cluster (which is also the center of the simulation domain), and **j**(**r**, *t*) is the microscopic current density obtained from the Kohn–Sham orbitals using the code octopus [48]. A detailed theoretical discussion can be found in Ref. 36. The frequency of the laser is chosen to be at LSPR (i.e., *ω*_*L*_ = *ω*_*p*_) of respective clusters as indicated by arrows in the spectra, and the duration of the pulse is set by choosing *n*_uni_ = 1. The temporal profiles of the *x* and *y* components of the generated orbital magnetic moment (i.e. M_*x*_(*t*) and M_*y*_(*t*)) are shown in the middle panel of Figure 1, while the bottom panel shows the same for M_*z*_(*t*) the *z*-component of **M**(*t*) for the three clusters.

For all the three clusters the induced orbital magnetic moment is found to be negligibly small along the directions (*x* and *y*) on the plane of polarization of the perturbing electric field: of the order of 10^−3^ *μ*_*B*_ for Na_7_, and 10^−5^ *μ*_*B*_ for Na_19_ and Ag_19_. The *z*-component of the induced orbital magnetic moments M_*z*_(*t*) in Na clusters increase with the duration of the laser pulse and oscillate around a non-negligible value: 0.3*μ*_*B*_ for Na_19_ and 0.12*μ*_*B*_ for Na_7_ when the laser is switched off. Similar temporal dependence of M_*z*_(*t*) was observed previously [36] in RT-TDDFT calculations on NPs modeled by jellium description which corroborates the generation of magnetic moment at LSPR in simple-metal clusters. In contrast to the jellium modeling, here the M_*z*_(*t*) in atomically precise clusters shows the presence of an oscillation after turning the laser off. This oscillation is due to the fact that in Na_7_ the laser field primarily excites the well-defined LSPR with very little overlap with the low-strength shoulders appearing on both sides of it. In Na_19_ the LSPR is built up by many energetically closely-spaced electronic transitions of comparable strength and the laser field is centered around the strongest of them with considerable overlap with the neighboring ones. This not only creates stronger oscillation in Na_19_ but also a slight decrease in the time-dependent M_*z*_(*t*) as compared to Na_7_ in which the oscillation lasts several tens of fs. As there is no energy-loss considered in the simulations the damping is entirely of Landau type.

In Ag clusters the electronic structures calculated using PBE XC functional show that the onset of *d*-electrons is about 3 eV below the Fermi level (cf. Figure S1 of the Supplementary Material). This is why for the Ag_19_ the LSPR, appearing at an energy higher than 3 eV, is strongly coupled with excitation involving *d*-electrons which are also driven by the laser field. Due to the highly localized nature of the *d*-electrons they respond to the dipolar surface mode of the LSPR and thus screen it significantly [46], [49]. For this very reason, and due to the strong coupling, the LSPR in Ag_19_ loses its plasmonic character and the generated M_*z*_(*t*) is significantly lower in Ag_19_ when compared to the Na clusters. At the same time, the localized *d*-electrons getting driven by the laser field follow the same periodicity of the laser in their localized circular motion around the ions. As discussed later, this gives rise to oscillation in M_*z*_(*t*) having the same frequency as that of the laser.

### 3.2 Simple and noble metals

**Figure 2:**
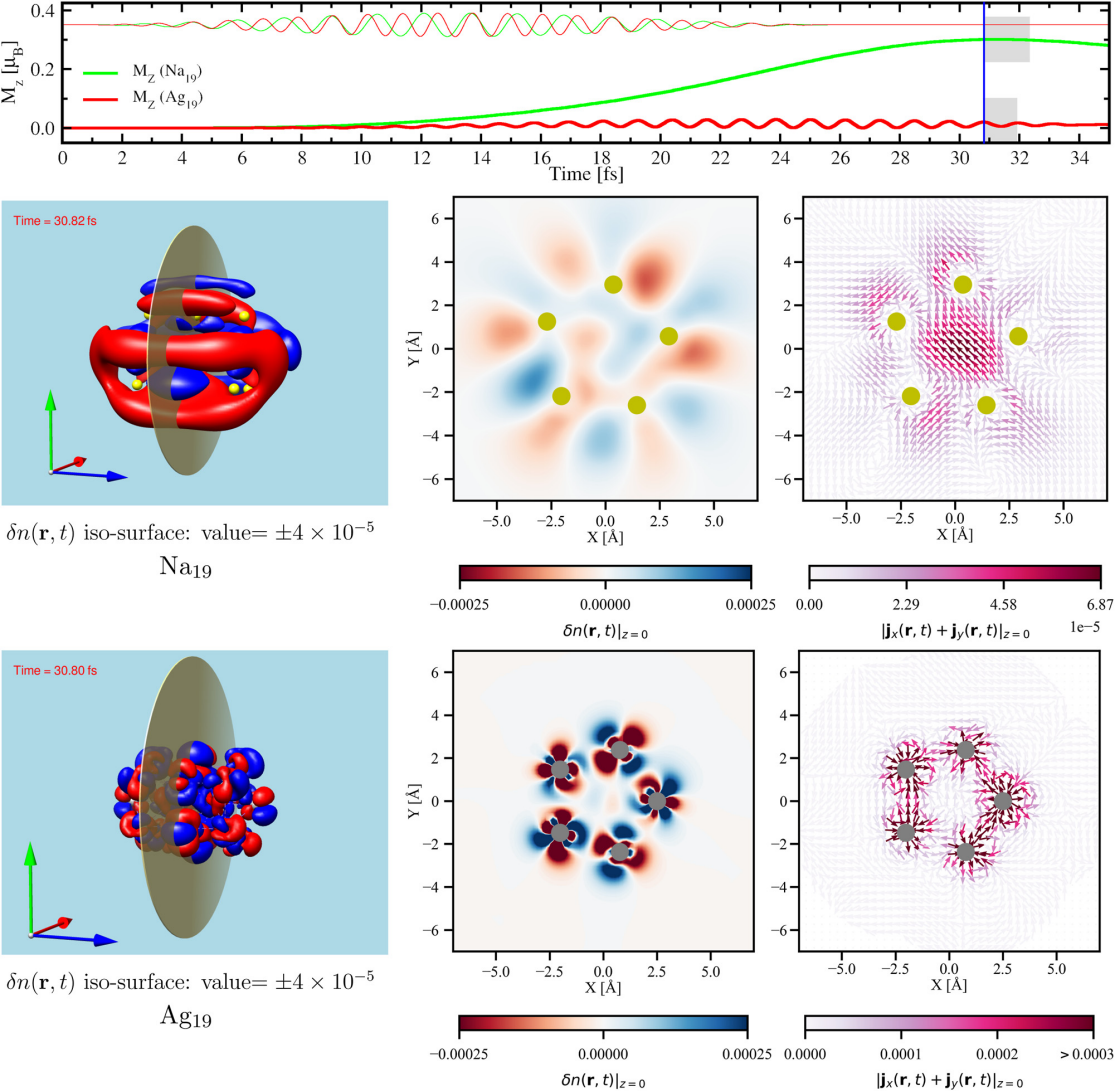
**Top**: thin lines – temporal profile of the *x*-component (*E*_*x*_) of the circularly polarized laser fields in arbitrary unit, used to excite Na_19_ (green) and Ag_19_ (red), and thick lines – the *z*-component of generated orbital magnetic moment (M_z_) same as the ones in Figure 1. The grey shaded regions on the two curves correspond to one time-period of the self-sustained circular motion of the induced density after the laser (of respective *ω*_*L*_ cf., Figure 1) is switched off. The blue line marks the time *t* ≈ 30.8 fs for which the induced density (*δn*) and the current density are shown in the middle and the bottom panels. **Middle** (Na_19_) and **bottom** (Ag_19_): The **left** panels show an iso-surface of the *δn* for iso-value $\pm 4\times 10^{-5}\;e{\overset{{}^{\circ}}{A}}^{-3}$ ; the **center** panels show color maps of *δn* for the XY-plane at *z* = 0 as indicated by the disks in the left panels. The **right** panels show the in-plane current density at *z* = 0: [**j**_*x*_ + **j**_*y*_](**r**, *t* ≈ 30.8 fs)|_*z*=0_ in $\mu_{B}{\overset{{}^{\circ}}{A}}^{-4}$ . The arrows show the direction, and the magnitude is represented by the color using the color map. The atoms are represented by balls and circles: yellow for Na and grey for Ag.

In order to understand the determining factors for differences in the generation of orbital magnetic moment in Na and Ag clusters having the same atomic arrangement and geometry we have looked into near-field quantities: the time-dependent induced density *δn*(**r**, *t*) and the current density **j**(**r**, *t*), in the self-sustained dynamics of the excited system i. e, after the laser is switched off. The results are presented in Figure 2. The top panel of the figure shows the *x*-component of the circularly polarized laser field, *E*_*x*_(*t*) (thin lines), and M_*z*_(*t*) for Na_19_ (green) and Ag_19_ (red). In the left panels, snapshots of the induced density *δn*(**r**, *t*) are shown as iso-surfaces for both the 19-atom clusters at around *t* = 30.8 fs marked by the blue line on the top panel of Figure 2. For the same instance of time, a cut through the *z* = 0 plane, indicated by the circular disk, shows the two-dimensional distribution of the *δn*(**r**, *t* ≈ 30.8 fs)|_*z*=0_ in the middle panels and of the in-plane current density [**j**_*x*_ + **j**_*y*_](**r**, *t* ≈ 30.8 fs)|_*z*=0_ in the right panels for the respective clusters. The directions of the current density vectors is represented by the arrows pivoted at midpoint on the real-space grid points, while the magnitudes are given by the color distribution represented through the color bar. The ion position is represented by balls and filled circles: yellow for Na and grey for Ag. The distribution of the induced density in the three-dimensional representation (left panels) and as shown by the two-dimensional color map (middle panels) clearly shows a strong dipolar contribution mostly coming from the surface in Na_19_ cluster. Under the action of the circularly polarized electric field there is a global rotation of the electron density about the *z*-axis in Na_19_ cluster as shown in a movie presented in Figure S3 of the Supplementary Material. This global rotation of the density having maximum contribution at the surface of the cluster is responsible for the creation of significant magnetization along the *z* direction. In contrast, in Ag_19_ the snapshots of the induced density and the current density both confirm strong spatial localization of the dynamics at regions around the ions. This is due to the fact that the laser having the frequency *ω*_*L*_ = 3.54 eV significantly excites the *d*-electrons (cf. Figure 1, & Figure S1) which are highly localized around the ions. On top of this ion-centered rotation of the *d*-electrons, there is a plasmon-driven global rotation of the electrons about the *z*-axis as shown in Figure S4 of the Supplementary Material containing movies of electron density and current density corresponding to one period of circular motion after the (circularly polarized) laser-field is switched off. As can be understood from the absorption spectrum (cf. Figure 1) and the density of states (cf. Figure S1) of Ag_19_, the cluster is more responsive for the *d*-electrons under the action of the laser with *ω*_*L*_ = 3.54 eV. This is why the atom-centered rotation of the *d*-electrons suppresses the global motion induced by the significantly damped plasmon, resulting in an oscillating magnetization which is much less significant than in the case of Na_19_. It is to be noted that this oscillation is different from the oscillations observed in the M_*z*_(*t*) in Na clusters. In Ag_19_, as the *d*-electrons get excited at their characteristic frequency (cf. Figure 1, & Figure S1) the oscillation in the M_*z*_(*t*) has the same frequency as the laser even after the laser is switched off.

**Figure 3:**
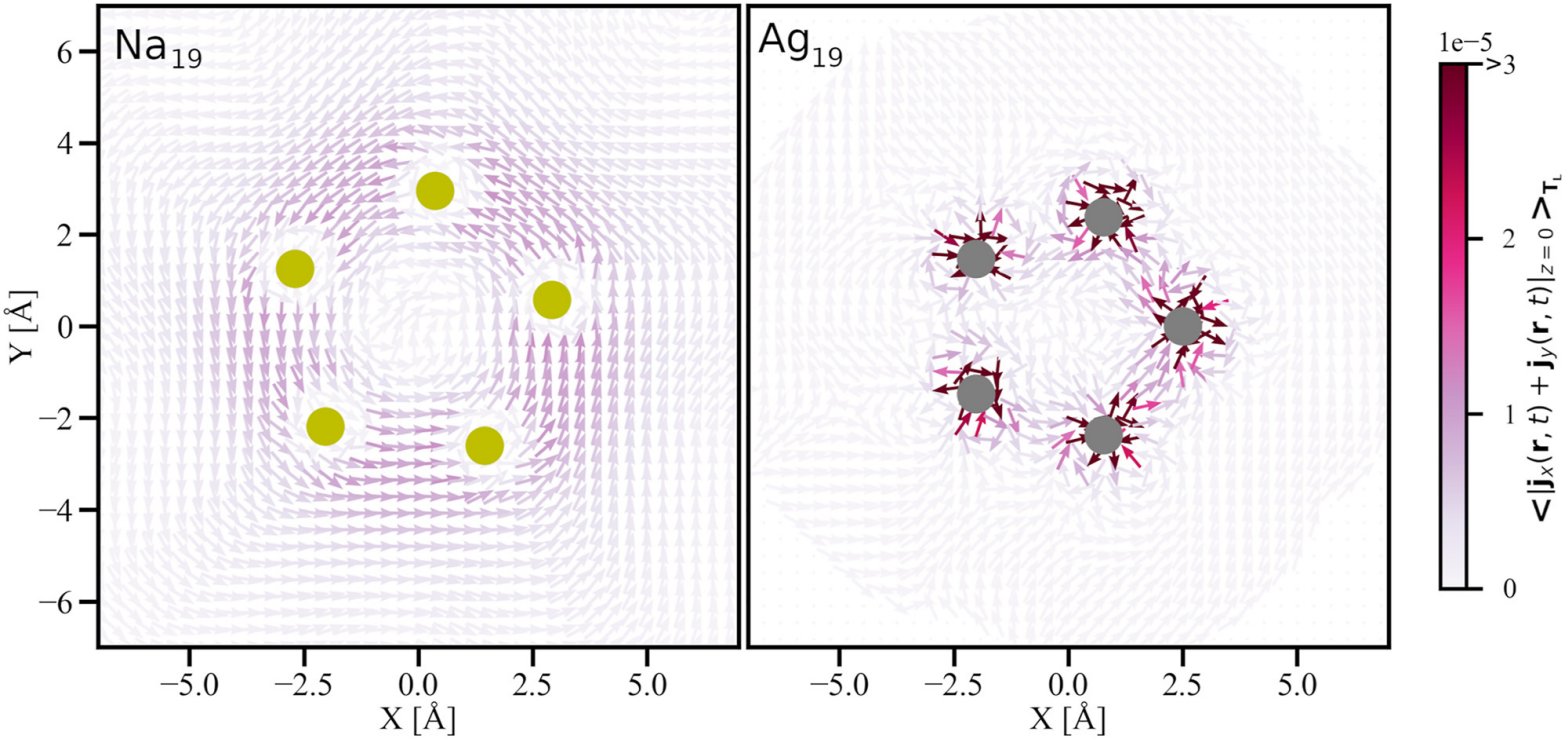
The in-plane current density at *z* = 0 in Na_19_ (**left**) and Ag_19_ (**right**) averaged over one period of circular motion of the electron density after the (circularly polarized) laser-field having respective LSPR frequencies is switched off. The arrows show the direction, and the magnitude is represented by the color using the color map. The atoms are represented by filled circles: yellow for Na and grey for Ag. The starting of the one-period circular motion is taken to be 30.8 fs marked by the blue line in Figure 2 and the duration of the period for each of the systems is represented by the width of the shaded grey regions on the same figure.

In order to better understand the spatial origin of the contributions responsible for the orbital magnetization, in Figure 3 we have looked into the in-plane current density at *z* = 0 in Na_19_ (left) and Ag_19_ (right) averaged over one period (*T*_*L*_) of circular motion of the electron density i.e., $<{\left[j_{x}\left(r,t\right)+j_{y}\left(r,t\right)\right]}_{z=0}{>}_{T_{L}}$ , after the (circularly polarized) laser-field is switched off. The starting of the one-period circular motion is taken to be 30.8 fs marked by the blue line in the top panel of Figure 2 and the duration of the period for each of the systems is represented by the width of the shaded grey regions. Following the fact that the distribution of the average current-density data is different in Ag_19_ than in Na_19_ (cf. Figure S6 of Supplementary Material), and in order to represent the average current density using the same color bar, we have chosen 0.0003 $\mu_{B}{\overset{{}^{\circ}}{A}}^{-4}$ as the maximum limit. All values of the average current density greater than this maximum limit are assigned the same color as for the maximum limit. The instantaneous current density as shown in Figure 2 shows only the spatial extent of charge flow. The one-period-averaged current density gives us information on the rotatory nature of the dynamics, i.e., whether or not the current forms a loop due to one full rotation of the electron density in the XY-plane creating a net magnetization along the *z* direction. This is clearly the case in Na_19_ cluster as can be evidenced in Figure 3. At the same time, the absence of a significant magnetization in Ag_19_ is also explained by the absence of the aforementioned current loop. Following the symmetry of the cluster about the *z*-axis and the color code we can also infer that the nonvanishing current loops in Na_19_ are stronger at the surface of the cluster than in its interior. This confirms the dominance of LSPR. In turn, on the one hand it shows the importance of exciting at LSPR energy and on the other hand it gives the possibility of exploiting the size- and species-dependent tunability of LSPR for the generation of light-induced magnetization due to IFE.

### 3.3 Plasmonic characteristics and the laser

**Figure 4:**
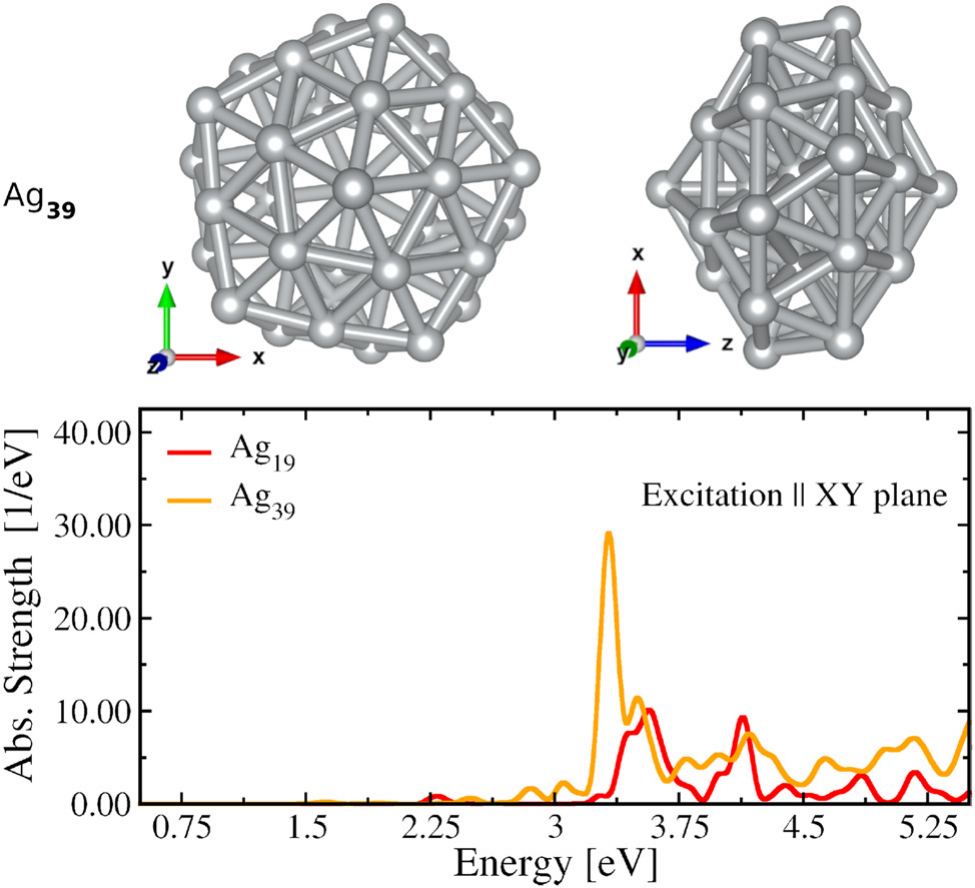
**Top**: the geometrical structure of Ag_39_ viewed from perpendicular to *z*-axis (left) and *y*-axis (right). **Bottom**: its absorption spectrum (orange) corresponding to excitation along the lateral direction (i.e. along the XY plane) compared with the same of Ag_19_ (red).

As compared to Na_19_ the generated magnetic moment in Ag_19_ is significantly low due to the lack of plasmonic characteristics. Therefore, we have explored another silver cluster, the Ag_39,_ in which the LSPR is well pronounced in the absorption spectrum. As shown by the comparison of absorption with Ag_19_ in the lower panel of Figure 4, for the same polarization of excitation, Ag_39_ possesses an LSPR peak at 3.32 eV which is well resolved in the spectrum and not dominated by the excitation involving *d*-electrons as the peak at 3.54 eV in the Ag_19_ spectrum. This is due to the fact that along the polarization of the excitation (i.e., the XY plane) Ag_39_ is more extended than Ag_19_, i.e., the lateral size of Ag_39_ is larger than that of Ag_19_. This gives the well-known sized-dependent red-shift of the LSPR (along the lateral direction) in Ag_39_ as compared to Ag_19_. This in turn allows the LSPR to decouple enough from the interband transitions involving *d*-electrons [44] to manifest itself in the spectrum.

**Figure 5:**
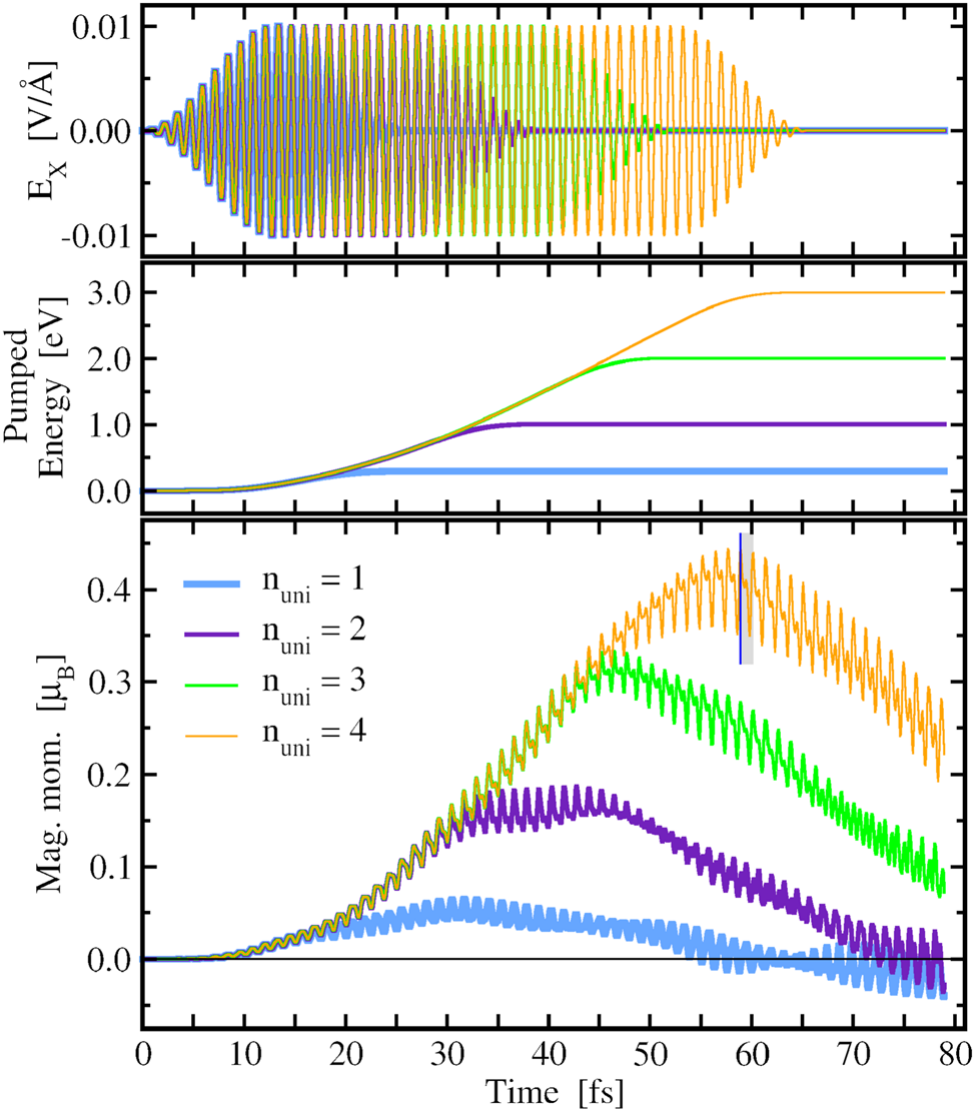
Effect of the duration of the circularly polarized laser pulse, as defined by *n*_uni_ in Eq. (2) (only *E*_*x*_(*t*) is shown in the **top** panel), on the generated orbital magnetic moment (**bottom** panel) in Ag_39_ cluster for *n*_uni_ varying from 1 to 4. The **middle** panel shows the time dependence of the total energy pumped into the system. The blue line marks the time *t* ≈ 58.9 fs for which the induced density and the current are shown in Figure 6. The grey shaded area corresponds to one period of circular motion of the laser-driven electron density.

Using the same polarization of the laser field as in the case of Ag_19_ we have excited the Ag_39_ cluster at its LSPR energy 3.32 eV. In order to investigate the effect of the pulse duration we have performed simulations for values of *n*_uni_ increasing from 1 to 4 (cf. Eq. (2)) representing increasing duration of the laser pulse. The results are shown in Figure 5. The upper, middle and lower panels respectively show the *x*-component of the laser *E*_*x*_(*t*), the total energy pumped into the system in course of time, and the M_*z*_(*t*) for *n*_uni_ = 1 to 4. We observe, first of all, that in comparison with Ag_19_ for *n*_uni_ = 1 (the red curve of Figure 2) we have a considerable amount of orbital magnetic moment M_*z*_ generated at the end of the laser pulse in Ag_39_ (the sky-blue curve of bottom panel of Figure 5). This confirms that the IFE is maximally effective at plasmon resonance, and as long as there is a well-defined LSPR peak in the absorption spectrum we can generate an orbital magnetic moment in silver clusters using a circularly polarized electric field. Furthermore, we can assert that the IFE-induced magnetization can be generated in a silver cluster even though the largest dimension of the cluster is of the order of 1 nm, which is the case for Ag_39_: the distance between two farthest atoms is 0.99 nm. While the duration of the laser increases with *n*_uni_ increasing from 1 to 4, we observe that the generation of the M_*z*_ also increases. This increase is directly proportional to the increase in the total energy pumped into the system as the laser keeps on exciting the system for longer duration. As both the IFE-induced M_*z*_ and the total pumped energy vary with the square of the electric field [36], the similarity between them as observed in Figure 5 confirms that the underlying mechanism behind the generation of M_*z*_ is IFE. Finally, it is worth noticing that no sooner the laser is switched off than the M_*z*_ starts to decrease with time. This fast decay is due to strong interference coming from the many energetically close transitions that constitute the LSPR in Ag_39_.

**Figure 6:**
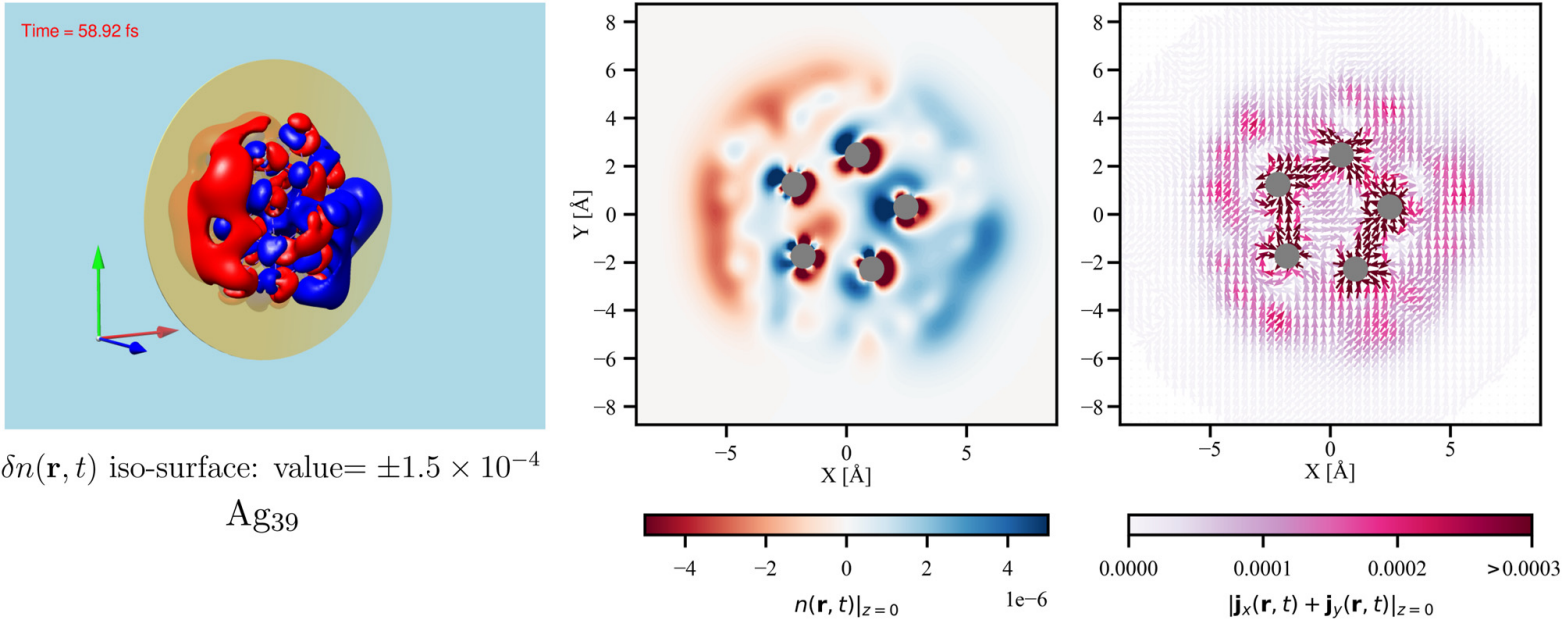
Ag_39_ cluster. The **left** panel shows an iso-surface of the *δn* for iso-value $\pm 1.5\times 10^{-4}\;e{\overset{{}^{\circ}}{A}}^{-3}$ ; the center panel shows *δn* for the XY-plane at *z* = 0 as indicated by the disks in the left panels. The **right** panel shows the in-plane current density at *z* = 0: [**j**_*x*_ + **j**_*y*_](**r**, *t* ≈ 58.9 fs)|_*z*=0_. The arrows show the direction, and the magnitude is represented by the color using the color map. The Ag atoms are represented by grey balls and circles.

In order to elucidate the plasmonic contribution in this generation of orbital magnetism in Ag_39_ we have looked into the laser-induced electron dynamics as presented in Figure 6.

Along with the atom-centered contributions from the *d*-electrons, the induced density in Ag_39_ shows significant contribution from the surface of the cluster, which was not seen in Ag_19_ (despite the fact that in Ag_19_ we looked into a lower iso-value to capture more spatial extension of the distribution). A comparison of the induced density in Ag_39_ and in Ag_19_ for same iso-values is shown in Figure S7 of the Supplementary Material. The dipolar surface contribution in Ag_39_ confirms the dominance of the plasmonic characteristics when the cluster is excited at *ω* = 3.32 eV. At the same time we observe that the polarity of the charge distribution around the atoms is exactly opposite to that at the surface. In other words, we see the well-known screening effect by the localized *d*-electrons in noble metal clusters [49]: the *d*-electrons are responding to the instantaneous electric field created by the LSPR. The instantaneous current density has strong contributions around the atoms similar to that observed in Ag_19_. However, it also shows uniform uni-directional contributions as we move radially away from the center of the cluster, which is not observed in Ag_19_. Under the action of the circularly polarized laser field these uni-directional arrows representing the direction of the instantaneous microscopic current density are found to rotate circularly pivoted at their mid-point changing their color signifying change of magnitude |**j**(**r**, *t*)|. This is shown in a movie presented in Figure S5 of the Supplementary Material. The motion of the arrows representing the atom-centered contributions is more complex as they come from the response of electrons having higher value of azimuthal quantum number.

**Figure 7:**
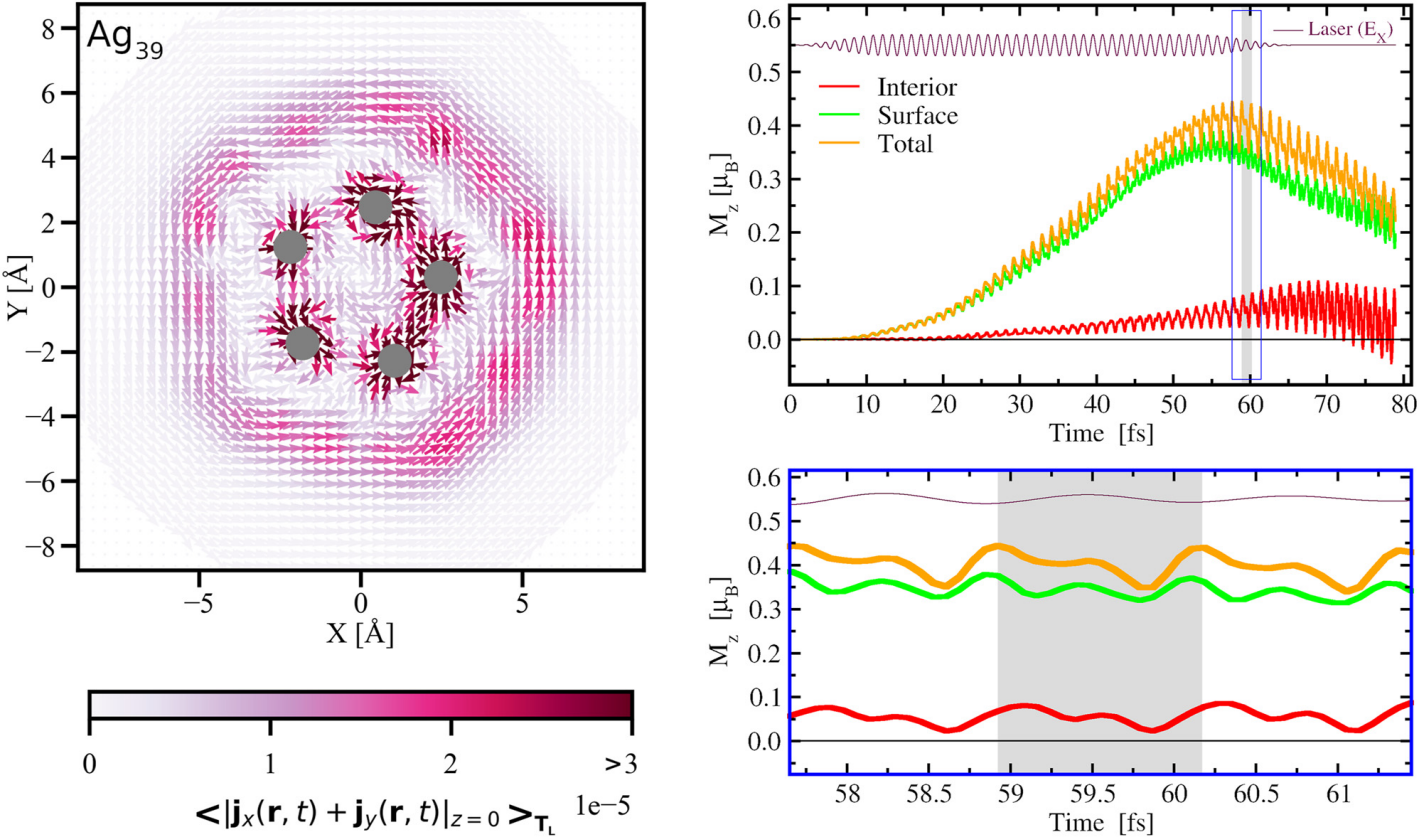
**L** **eft**: the in-plane current density at *z* = 0 in Ag_39_ averaged over one period (shaded in grey in right panels) of circular motion of the electron density corresponding to excitation by circularly polarized laser-field having *ω*_*L*_ = *ω*_*p*_ = 3.32 eV and *n*_uni_ = 4. The arrows show the direction, and the magnitude is represented by the color using the color map. The atoms are represented by balls and circles: yellow for Na and grey for Ag. **Right**: contributions from the interior (red) and the surface (green) of the cluster which add up to the total (orange) M_*z*_ as represented in Figure 5. *E*_*x*_(*t*) in arbitrary units is shown for reference. The **bottom** panel is an enlargement of the blue box in the **top** panel, and the black horizontal lines indicate the zero of M_*z*_.

Further insights on the spatial contributions on the generated magnetic moment in Ag_39_ are obtained from the one-period-averaged current density in Figure 7. The left panel shows the in-plane component of this averaged current density at *z* = 0 i.e., $<{\left[j_{x}\left(r,t\right)+j_{y}\left(r,t\right)\right]}_{z=0}{>}_{T_{L}}$ . The period *T*_*L*_ = 2*π*/*ω*_*L*_ over which the current is averaged is 1.245 fs. It is chosen around the maximum value of the generated M_*z*_ as shown by the grey shaded region in the right panels. In this period-averaged representation we clearly notice a current circulating at the surface of the cluster similar to the one in Na_19_ confirming the dominant contribution of the LSPR. At the same time, this near-field picture clearly shows the formation of concentric current loops perpendicular to the *z*-direction responsible for the generation of M_*z*_. The quantum mechanical nature of the excitation is corroborated by the spatial separation of this surface contribution from the contributions localized around the interior atoms, which come from the *d*-electrons. We have calculated the contribution to M_*z*_ coming from the surface by considering contribution outside a cylinder of radius $\rho =\sqrt{x^{2}+y^{2}}$ with its axis passing through the center of the cluster along the *z*-direction. The contributions inside (outside) this radius give the contribution to M_*z*_ coming from the interior (surface) of the cluster. The time dependence of these contributions for *ρ* = 3.6 Å, is shown in the right panels of Figure 7: surface in green, interior in red and the total M_*z*_ in orange as represented in Figure 5. The *x*-component of the laser is also shown in arbitrary unit for reference. The bottom panel shows an enlargement containing three periods of laser oscillation including the one (shaded grey area) for which the average current is calculated.

In the two-dimensional representation of the current at *z* = 0 we observe the atoms (represented by grey spots) only from the interior but not at the surface because at *z* = 0 there are none at the surface of the Ag_39_ cluster. However, there are 20 silver atoms which constitute the outer surface of the cluster perpendicular to the *z*-axis. The contribution from the *d*-electrons of these surface atoms also merges with the LSPR contribution creating oscillatory behavior of the M_*z*_, as seen on the green curves of Figure 7, having a periodicity same as that of the driving field. We note that the period of averaging is the same as that of the laser field. This green curve also confirms that it is the surface contribution which brings the maximal weight in the generation of M_*z*_, even in this silver cluster of 1 nm size. We also observe laser-driven oscillations in the red curve, which represents the contribution to M_*z*_ from the interior of the cluster. Like in Ag_19_, this is due to the atom-centered current originating from localized *d*-electrons. The red curve remains quite flat, very similar to the one observed for Ag_19_. It confirms that the interior of the cluster contributes very little to the generation of M_*z*_.

## 4 Conclusions

The results of the ab-initio calculations presented in this work confirm that IFE-driven orbital magnetic moment can be induced in simple- and noble-metal clusters when excited at the LSPR frequency. We have shown that, when excited with circular polarization and at the plasmon resonance energy, a significant magnetic moment can be generated in the sodium cluster Na_19_ but also in a cluster as small as Na_7_. Although not explicitly shown, it is worth mentioning that a laser tuned to a higher excitation energy would give rise to a magnetization proportional to the absorption strength as long as the excited electrons are delocalized and correspond to collective (plasmonic) surface mode of absorption. This is because under the action of a circularly-polarized laser the excited electrons will form concentric current loops (and not atom-centered ones). While this would be the case for Na_19_, in Ag_19_ already the first absorption peak is significantly suppressed by the *d*-electron excitations which are spatially localized. Therefore, despite having the same number of atoms and the same geometry and structure the 19-atom cluster of silver did not produce an orbital magnetic moment as significant as its sodium counterpart. At a higher excitation energy in Ag_19_ (e.g., around 4.0 eV) we have more contribution (if not 100 %) of the *d*-electrons. Therefore, circularly-polarized laser-excitation at a higher energy in Ag_19_ will give a similar or even less orbital magnetic moment. However, thanks to its disk-like flat geometry, Ag_39_ shows a well-resolved LSPR in the absorption spectrum which is not drowned by the interband transitions involving the *d*-electrons. When excited with a circularly polarized laser at this LSPR energy, in Ag_39_ we obtained a noticeable orbital magnetic moment. This confirms that while using a circularly polarized laser pulse with the frequency of the LSPR, the decisive criterion to generate a significant IFE-driven orbital magnetic moment in noble-metal clusters is that the LSPR should be well-resolved in the spectrum and dominant over the interband transitions involving *d*-electrons. For different pulse duration of the laser in Ag_39_, the LSPR-induced magnetic moment varies almost linearly with the total energy pumped by the laser. This is in accordance with previous results [36] on NPs described using jellium model and confirms that the underlying mechanism is indeed the inverse Faraday effect. Further confirmation of the importance of exciting at LSPR is gained from the spatially resolved near-field analysis. The time-dependent induced density and the current density averaged over one period of laser-induced circular motion of the electrons revealed that the orbital magnetic moment is generated principally by the rotating dipolar contribution of the surface mode coming from the LSPR. The analysis also confirmed that, for the clusters considered in this work, the interior of the cluster contributes very little. Unlike in sodium, for the case of silver clusters the generated orbital magnetic moment displays an oscillatory behavior. This is due to the localized *d*-electrons, inherent to silver systems, which respond to the dynamic electric field created by the dipolar LSPR surface mode giving rise to a complex atom-centered rotatory dynamics having the same frequency as of the laser field. These atom-centered contributions of *d*-electrons coming from the surface atoms add up to the principal dipolar contribution of the surface mode of the LSPR and give the oscillatory behavior of orbital magnetic moment. Finally, it is worth mentioning that this LSPR-induced creation of magnetization occurs on an ultrafast timescale: an orbital magnetic moment of $\approx 0.3\mu_{B}$ in Na_19_ and $\approx 0.4\mu_{B}$ in Ag_39_ could be generated in, respectively, ≈32 fs (cf. Figures 1 and 2) and ≈57 fs (cf. Figure 5) which are at least two orders of magnitude faster than the speed of magnetic switching in present-day materials [50], [51]. The ab-initio results presented in this work unravel the knowledge of the dependence of orbital magnetic moment on the geometry and the constituent species of the metal clusters, as well as its dependence on the laser field. At the same time, they confirm that plasmonic metal clusters can be the building block of ultrafast all-optical switching, and thus predict the possibility of metal-cluster-based nanometric systems for applications in ultrafast magnetic switching.

## Associated content

Supplementary Material contains the electronic structures and laser-driven real-time dynamics of the excited clusters.

## Supplementary Material

Supplementary Material Details
