# Supplementary material for: Orbital magnetism through inverse Faraday effect in metal clusters
Source: Nanophotonics. 2024 Sep 16;13(23):4291–302. doi: 10.1515/nanoph-2024-0352 (PMC11636370; doi:10.1515/nanoph-2024-0352)
Supplement: Supplementary file 1 — Supplementary Material Details [file j_nanoph-2024-0352_suppl_001.pdf]

Supplementary Material: Orbital Magnetism Through Inverse Faraday Effect in Metal Clusters

Deru Lian,† Yanji Yang,† Giovanni Manfredi,‡ Paul-Antoine Hervieux,‡ and Rajarshi Sinha-Roy*,†

†*Universite Claude Bernard Lyon 1, CNRS, Institut Lumière Matière, UMR5306, F-69100, Villeurbanne, France*

‡*Université de Strasbourg, CNRS, Institut de Physique et Chimie des Matériaux de Strasbourg, UMR 7504, F-67000 Strasbourg, France*

E-mail: rajarshi.sinha-roy@univ-lyon1.fr

For visualization

Please use any of the two following pdf readers in order to visualize the movies of the dynamics of the excitation:

1) Okular (open source),

2) Adobe acrobat reader .

Other pdf readers show only the first frame of the movies as an image.

Density of states of Ag_{19} and Ag_{39}

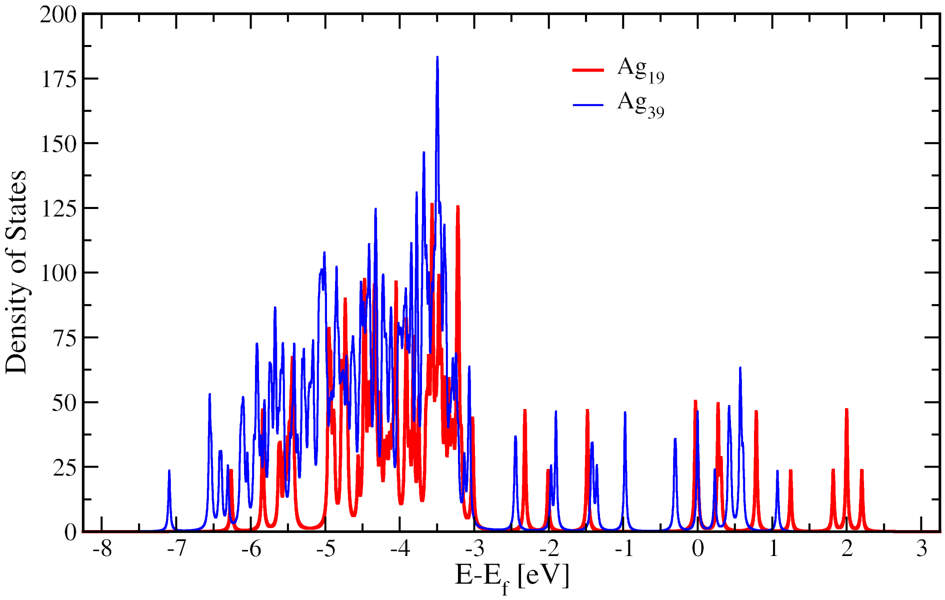

Fig. S1: The density of states (DOS) in both the silver clusters (Ag_{19} and Ag_{39}) show that the onset of the *d*-electrons in the electronic structure of the clusters takes place at around 3 eV below the Fermi level.

Temporal profile of the circularly polarized laser

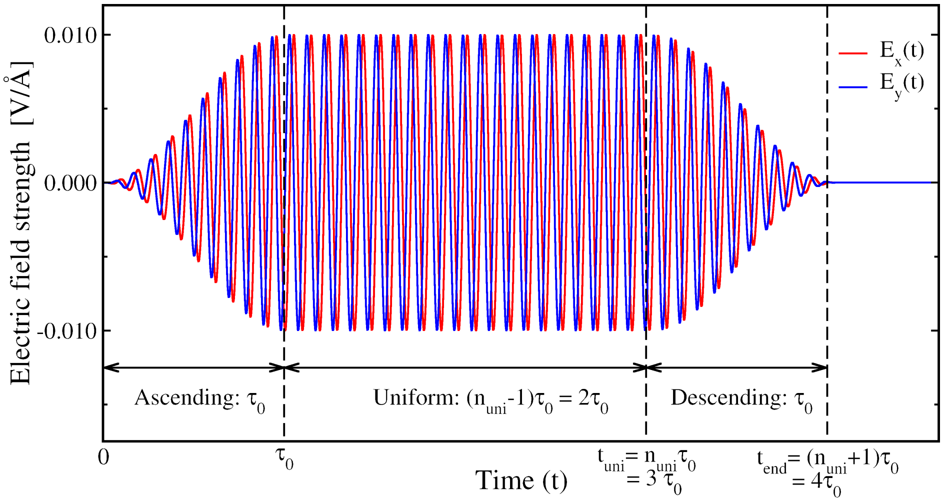

Fig. S2: The temporal profile of the $x-$ (red) and $y-$ (blue) components of the spatially homogeneous circularly polarized laser field corresponding to $n_{uni} = 3$ as described in Equations 1 (Eq. 1 in main text) and 2 (Eq. 2 in main text).

As mentioned in the main text of the article, the circularly polarized laser field can, in general, be described as

$$\mathbf{E}(t) = E_x(t)\hat{\mathbf{x}} + E_y(t)\hat{\mathbf{y}} = F(t)\left[cos(\omega_{_L} t)\hat{\mathbf{x}} + cos(\omega_{_L} t - \frac{\pi}{2})\hat{\mathbf{y}}\right] \tag{1}$$

where,

$$F(t) = E_0 \begin{cases} sin^2\left(\dfrac{\pi}{2\tau_0} t\right) \text{ for } t \leq \tau_0; \\ 1, \text{for } \tau_0 < t \leq t_{uni}, \text{ where } t_{uni} = n_{uni}\tau_0; \\ cos^2\left(\dfrac{\pi}{2\tau_0}\ (t - t_{uni})\right), \text{for } t_{uni} < t \leq t_{end}, \text{ where, } t_{end} = t_{uni} + \tau_0; \\ 0, \text{ for } t > t_{end}. \end{cases} \tag{2}$$

Here, τ_0 is chosen to be $20\hbar/\text{eV}$ (≈ 13 fs), and $\omega_{_L}$ represents the energy of the laser field. The maximum amplitude is $E_0 = 10^{-2}$ V/Å; $t_{\text{uni}} = n_{\text{uni}}\tau_0$, where n_{uni} is an integer and determines the constant-in-time segment of the envelope. The duration of the ascending segment of the

pulse is τ_0, the same is for the descending segment, and the duration of the uniform segment is $(n_{\text{uni}}-1)\tau_0$, which makes the total duration of the laser pulse $(n_{\text{uni}}+1)\tau_0$. As shown in the Equation 1, the plane of polarization of the electric field is along the XY plane (i.e., same as for the spectra).

Circularly-polarized laser induced dynamics

Na_{19} cluster: $\omega_L = \omega_p = 2.65$ eV

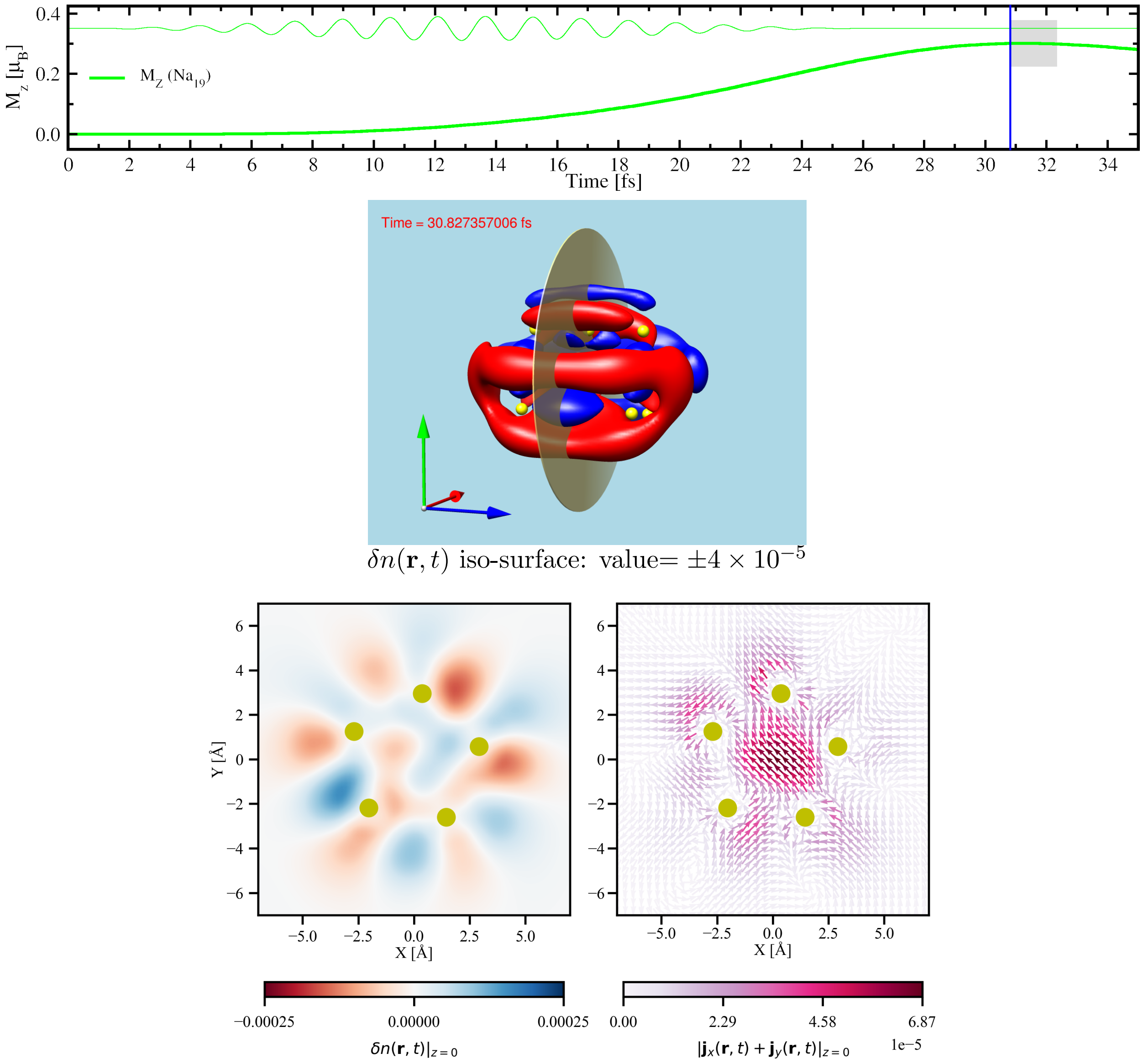

Fig. S3: **Top**: thin line – temporal profile of the x-component (E_x)of the circularly polarized laser fields in arbitrary unit used to excite Na_{19}, and thick line – the z-component of generated orbital magnetic moment (M_Z) same as the ones in Figure 1. The grey shaded region correspond to one full period of the self-sustained circular motion of the induced density after the laser is switched-off. The blue line marks $t \approx 30.8$ fs, the starting time of the period for which the induced density (δn) and the current are shown. The **middle** panel shows an iso-surface of the δn for iso-value $\pm 4 \times 10^{-5}$ $e\text{Å}^{-3}$; the **bottom-left** panel shows δn for the XY-plane at $z = 0$ as indicated by the disk in the middle panel. The **bottom-right** panel shows the in-plane current at $z = 0$: $[\mathbf{j}_x + \mathbf{j}_y](\mathbf{r}, t \approx 30.8 \text{ fs})|_{z=0}$ in $\mu_B \text{Å}^{-4}$. The arrows show the direction and the magnitude is represented by the color using the color map.

$\mathbf{Ag_{19}}$ cluster: $\omega_L = \omega_p = 3.54$ eV

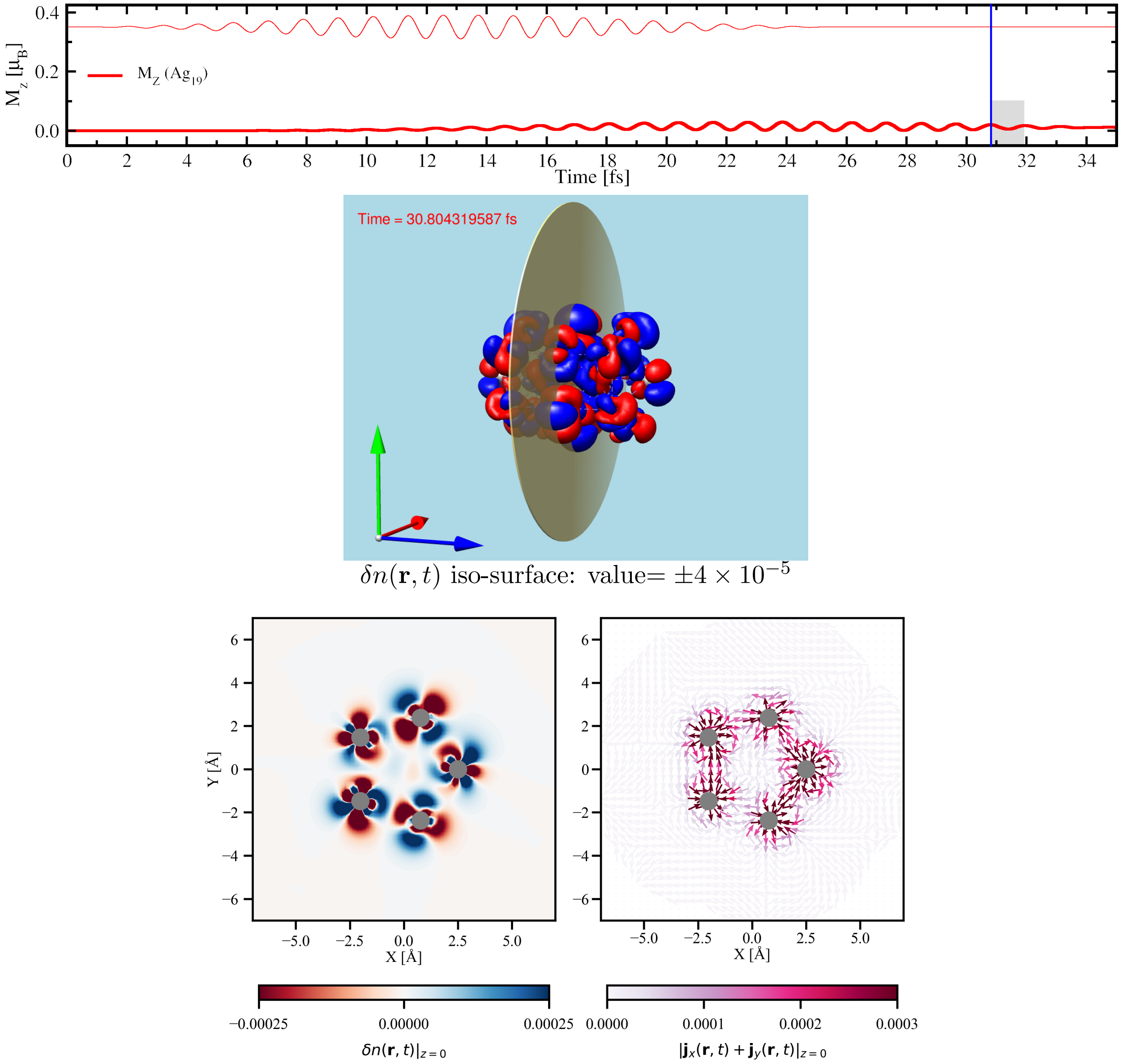

Fig. S4: **Top**: thin line – temporal profile of the x-component (E_x)of the circularly polarized laser fields in arbitrary unit used to excite Ag_{19}, and thick line – the z-component of generated orbital magnetic moment (M_Z) same as the ones in Figure 1. The grey shaded region correspond to one full period of the self-sustained circular motion of the induced density after the laser is switched-off. The blue line marks $t \approx 30.8$ fs, the starting time of the period for which the induced density (δn) and the current are shown. The **middle** panel shows an iso-surface of the δn for iso-value $\pm 4 \times 10^{-5}$ $e\text{Å}^{-3}$; the **bottom-left** panel shows δn for the XY-plane at $z = 0$ as indicated by the disk in the middle panel. The **bottom-right** panel shows the in-plane current at $z = 0$: $[\mathbf{j}_x + \mathbf{j}_y](\mathbf{r}, t \approx 30.8 \text{ fs})|_{z=0}$ in $\mu_B\text{Å}^{-4}$. The arrows show the direction and the magnitude is represented by the color using the color map.

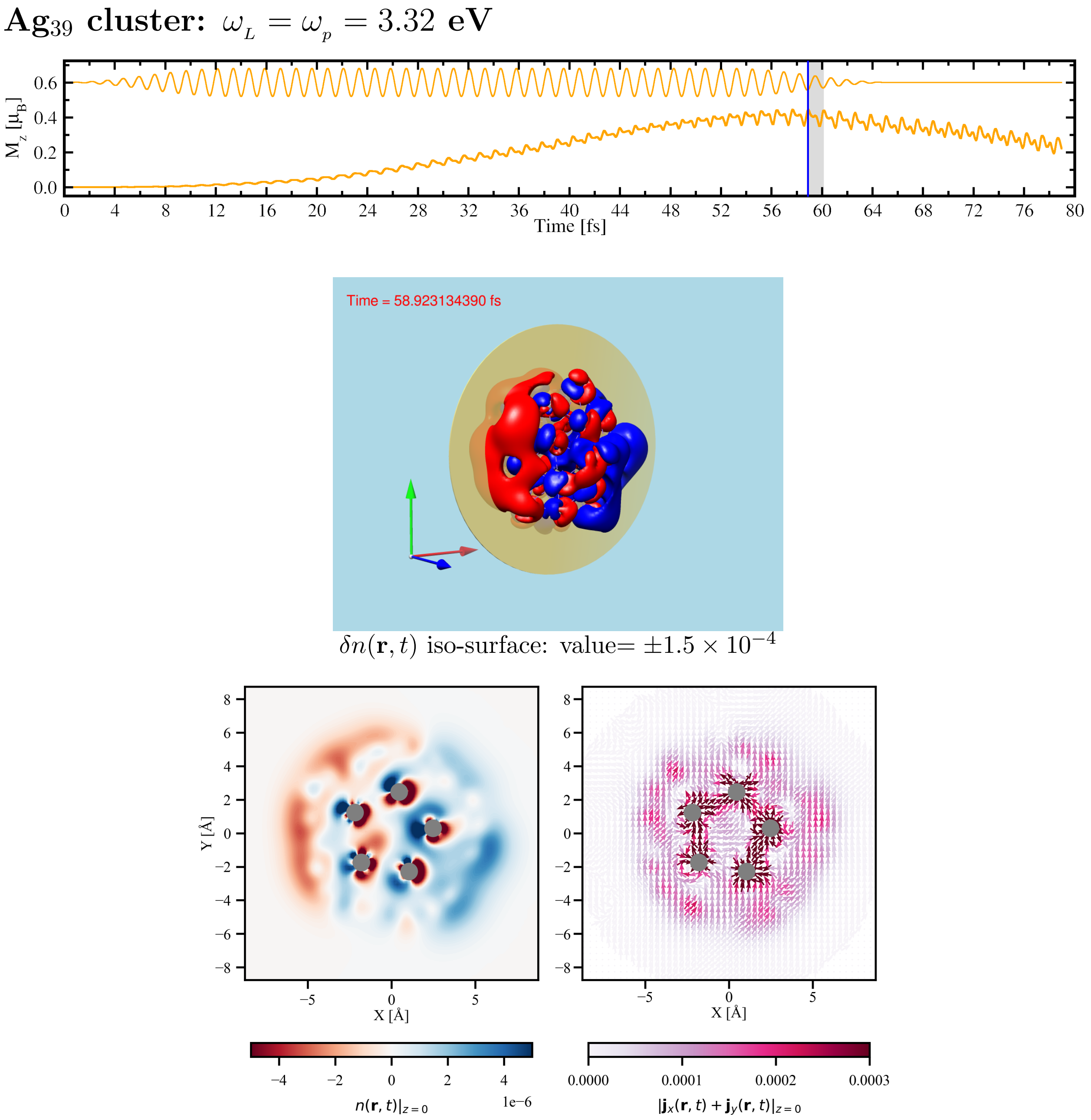

Fig. S5: **Top**: thin line – temporal profile of the x-component (E_x)of the circularly polarized laser fields in arbitrary unit used to excite Ag_{39}, and thick line – the z-component of generated orbital magnetic moment (M_Z) same as the ones in Figure 1. The grey shaded region correspond to one full period of the self-sustained circular motion of the induced density after the laser is switched-off. The blue line marks $t \approx 58.9$ fs, the starting time of the period for which the induced density (δn) and the current are shown. The **middle** panel shows an iso-surface of the δn for iso-value $\pm 1.5 \times 10^{-4}$ $e\text{Å}^{-3}$; the **bottom-left** panel shows δn for the XY-plane at $z = 0$ as indicated by the disk in the middle panel. The **bottom-right** panel shows the in-plane current at $z = 0$: $[\mathbf{j}_x + \mathbf{j}_y](\mathbf{r}, t \approx 58.9 \text{ fs})|_{z=0}$ in $\mu_B\text{Å}^{-4}$. The arrows show the direction and the magnitude is represented by the color using the color map.

Histograms of the data for current densities in Na_{19}, Ag_{19}, & Ag_{39} averaged over one period

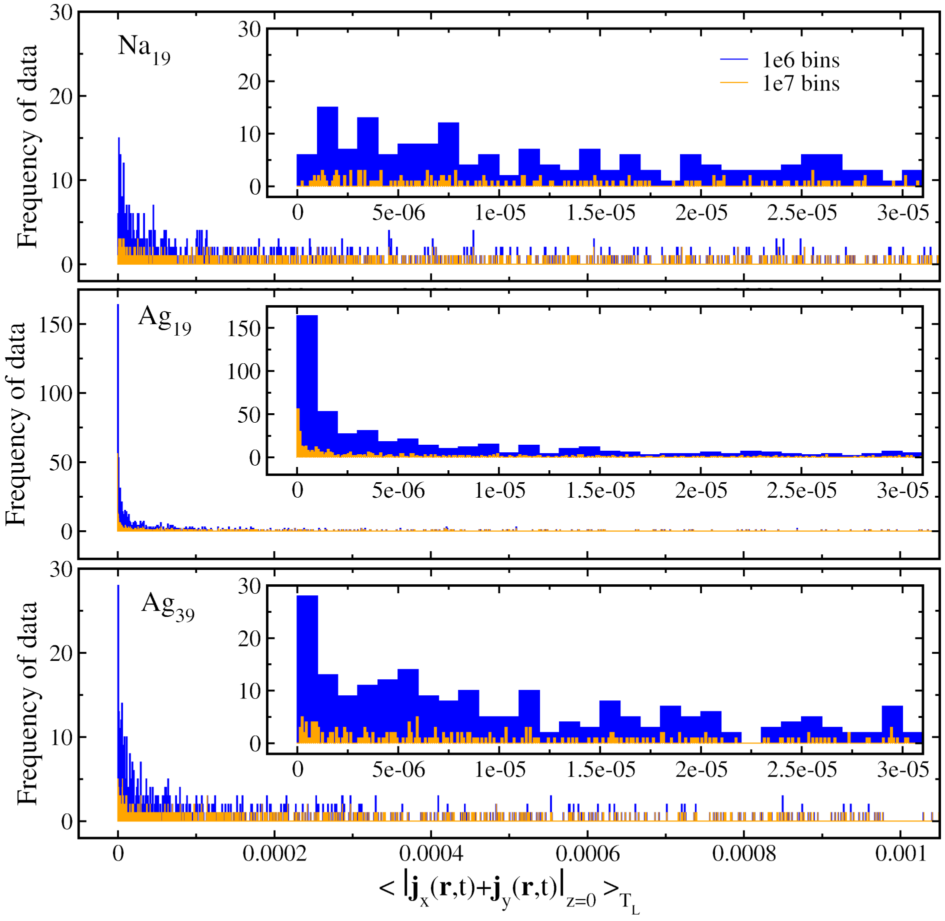

Fig. S6: Histograms created with two different binning (10^6 in blue and 10^6 in orange) represent the data corresponding to the magnitude of the current density averaged over one full period of circular motion in Na_{19} (**top**), Ag_{19} (**middle**), and Ag_{39} (**bottom**). The insets are zoom of graphs from values 0 to 3×10^{-5}.

The histograms for Ag_{19} in Figure S6 reveal that most of the data are below the value 10^{-5}. It also shows that the data frequency in Ag_{19} decreases with value more rapidly than in Na_{19}. For this very reason, representing the data for these two clusters using the same color bar is challenging. Comparing the two different scales of the distribution of the data we find that the scale having the maximum limit of 3×10^{-5} is more adapted to represent the two datasets (i.e., for Na_{19} & Ag_{19}) that the scale having the maximum limit of 10^{-3}.

Induced density ($\delta n(\mathbf{r},t)$) for the same iso-values

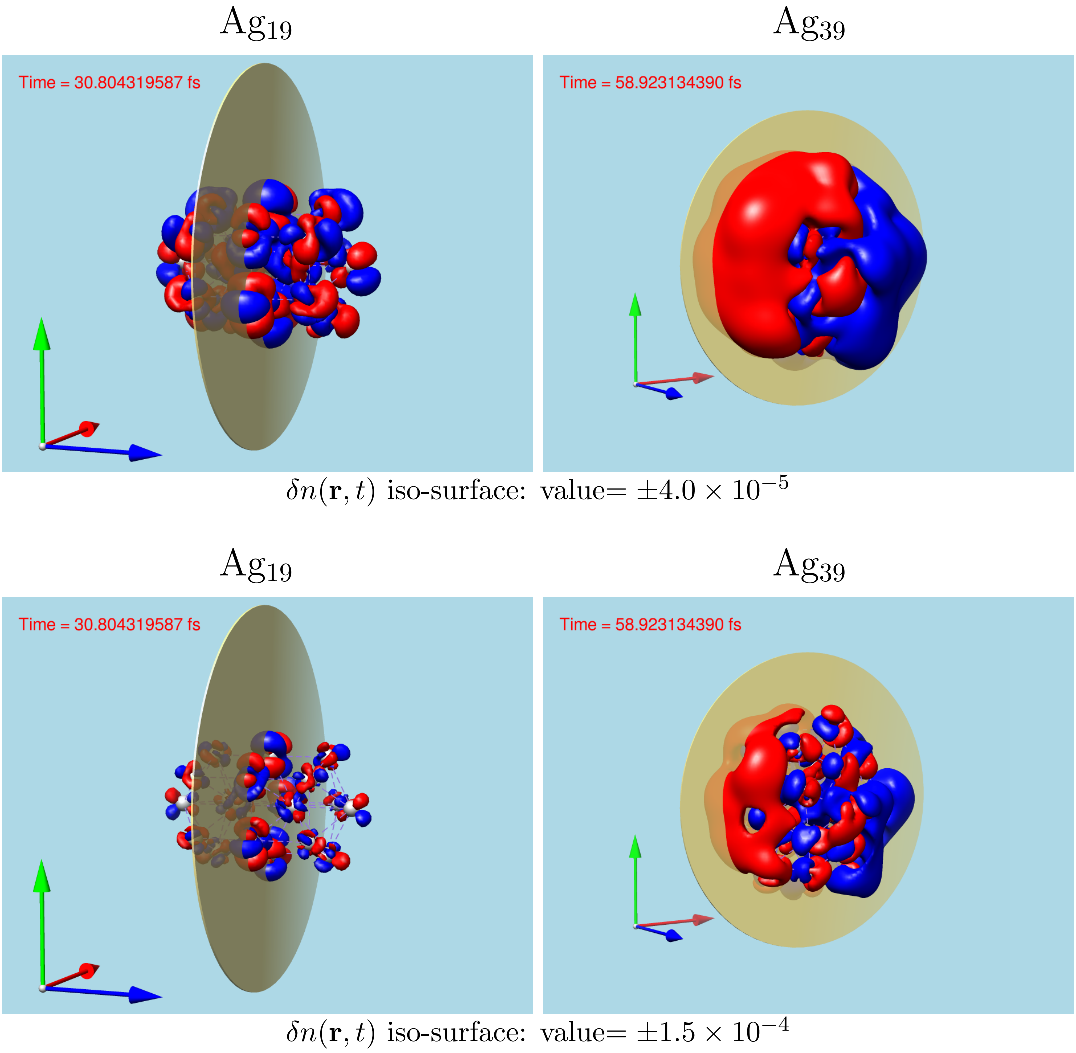

Fig. S7: Induced densities corresponding to laser excitation in Ag_{19} (left) and Ag_{39} (right) clusters are compared for the same values of iso-surface: 4.0×10^{-5} (top) and 1.5×10^{-4} (bottom).

As we observe in the comparison of $\delta n(\mathbf{r},t)$ depicted in Figure S7, the lower the iso-value more spatially extended is the iso-surface. In the main article we show the $\delta n(\mathbf{r},t)$ in Ag_{19} in Figure 2 for lower iso-value $= \pm 4.0 \times 10^{-5}$ (the top left panel of Figure S7). For Ag_{39} the $\delta n(\mathbf{r},t)$ is shown in Figure 6 for higher iso-value $= \pm 1.5 \times 10^{-4}$ (the bottom right panel of Figure S7). Here the comparison of both in Figure S7 using the same iso-value confirms that the inferences made in the main article holds: there is no surface contribution in Ag_{19}, and in Ag_{39} the surface contribution dominates.
